# Assessing the threat of artemisinin resistance: seasonal and ecological patterns of *PfKelch13* mutation distribution in malaria-endemic communities in Ghana

**DOI:** 10.3389/fcimb.2026.1846022

**Published:** 2026-09-25

**Authors:** Mavis Puopelle Dakorah, Enoch Aninagyei, Godwin Adzakpah, Juliana Attoh, Isaac Tukwarlba, Desmond Omane Acheampong

**Affiliations:** 1 Department of Medical Laboratory Sciences, School of Allied Health Science, University of Cape Coast, Cape Coast, Ghana; 2 Department of Biomedical Sciences, University of Health and Allied Sciences, Ho, Ghana; 3 Department of Health Information Management, School of Allied Health Science, University of Cape Coast, Cape Coast, Ghana; 4 Department of Biomedical Sciences, School of Allied Health Science, University of Cape Coast, Cape Coast, Ghana

**Keywords:** artemisinin, epidemiology, *Pfkelch13*, Plasmodium falciparum, resistance

## Abstract

**Background:**

Resistance to Artemisinin-based combination therapies (ACTs) threatens the backbone of Ghana’s success in Malaria control. In addition, malaria remains prevalent in the Central Region, raising concerns about the potential emergence of drug resistance. Mutations in the *PfKelch13* gene associated with reduced susceptibility have been reported across Africa and in some parts of Ghana, highlighting the need for molecular surveillance in these endemic settings.

**Method:**

Dried blood spots were prepared from whole blood, and genomic DNA was amplified using established *Plasmodium*–specific primers. Selective whole-genome amplification enriched *Plasmodium falciparum* DNA, and libraries were sequenced using high-throughput Illumina HiSeq 2500 technology.

**Results:**

358 samples were successfully sequenced. *PfKelch13* wild-type (WT) allele remained predominant, ranging from 42% to 100%, but the burden and diversity of mutant alleles differed substantially by location. SNPs observed were 127 single mutations, 42 double mutations, 2 Triple mutations, and 1 Quadruple mutation. Neither the ecological zone nor the season was significantly associated with the occurrence of mutant haplotypes; however, the district of origin emerged as statistically significant.

**Conclusion:**

The study demonstrates the presence of *PfKelch13* mutations in *Plasmodium falciparum* in the Central Region of Ghana, with substantial genetic diversity but low frequencies of validated resistance markers. The spatial and seasonal heterogeneity observed across transmission settings suggests that factors operating at finer geographical and temporal scales may be important determinants of genetic variation, highlighting limitations of broad ecological classifications. These findings underscore the need for continuous, fine-scale molecular surveillance and therapeutic efficacy studies to detect and mitigate emerging artemisinin resistance proactively.

## Background

The main first-line treatments of uncomplicated malaria in Ghana since 2006 have been artemether-lumefantrine, artesunate-amodiaquine and dihydroartemisinic-piperaquine (MOH and Ministry of Health,Ghana, 2009). Agreeably, the integration of ACTs (combining artemisinin derivatives with partner drugs) into global health protocols has facilitated patient cure and the clearance of both asexual and sexual parasite stages. These therapies interrupt the transmission cycle to the vector (Rodrigues et al., 2022), contributing to the worldwide reduction in malaria morbidity and mortality and advancing the goal of malaria elimination (WHO, 2020). However, malaria prevalence remains high in some areas of Ghana, including the Central Region, despite the long-standing availability of these therapies (GSS and Ghana Statistical Service, 2017; Dakorah et al., 2022).

These ACTs combine fast-acting artemisinin with slower-acting partner drugs like lumefantrine or mefloquine to maximise efficacy. While artemisinin can achieve a 10,000-fold reduction in parasite density within one 48-hour cycle, the partner drug persists longer to clear remaining parasites and support sustained treatment effectiveness (Stokes et al., 2021). Extensive genomic research has identified various SNPs in the *PfKelch13* gene that are responsible for artemisinin resistance (Eboumbou Moukoko et al., 2019; Ishengoma et al., 2019; Imwong et al., 2020; Dieng et al., 2023). The mutations in the *PfKelch13* gene are suspected to hinder haemoglobin endocytosis by ring-stage parasites, thereby slowing the rate of parasite clearance following therapy (Yang et al., 2019; Birnbaum et al., 2020).

The WHO classifies *PfKelch13* SNPs as “validated resistance markers” if they are linked to slow parasite clearance and exhibit >1% survival in ring-stage assays. Only those mutations which satisfied one criterion were called “candidate markers”, and those which satisfied neither were called “neutral” (Ménard et al., 2016). Validated *PfKelch13* artemisinin resistance markers are Y493H, R539T, C580Y, F446I, N458Y, P574L, A675V, M476I, I543T, P553L, C469Y, R622I and R561H. Meanwhile, mutations such as P441L, G449A, C469F, A481V, R515K, P527H, N537I/D, G538V and V568G are considered candidate resistance markers (Ikegbunam et al., 2022; Azmi et al., 2023). The frequencies of these resistance markers are significantly higher in Southeast Asia than in Africa (Bwire et al., 2020).

Resistance markers for *PfKelch13* have been validated in Africa, including R622I in Eritrea and A675V and C469Y in Uganda. R561H and P441L have also been described in Tanzania, Uganda, Rwanda and the Democratic Republic of the Congo (DRC) (Balikagala et al., 2021; Uwimana et al., 2021; Conrad et al., 2023; Juliano et al., 2023; Mihreteab et al., 2023; Kahunu et al., 2024). The C580Y SNP was first reported in Chinese migrants in Ghana in 2013 (Feng et al., 2015) and later found among blood donors (Aninagyei et al., 2020). Other validated markers, such as M476I and Y493H, have been reported in independent studies in Ghanaian *P. falciparum* parasites in more recent years (Matrevi et al., 2019; Dieng et al., 2023). Furthermore, clinical findings from the Northern regions of Ghana have documented instances of delayed parasite clearance (Abuaku et al., 2021).

These recent findings suggest that artemisinin resistance might already be present but unnoticed in Ghana. This makes the country a likely candidate for the evolution of new resistance forms and highlights the importance of continuous monitoring of parasite populations, especially in highly endemic regions like the Central Region. This cross-sectional study investigated *PfKelch13* mutations in *P. falciparum* from selected districts in the Central Region. The research sought to evaluate whether artemisinin gene SNPs in this malaria-prevalent setting accounting for the impact of existing control measures and the region’s history of antimalarial use.

## Materials and methods

### Study design

This was a multi-centre cross-sectional study in five study sites in the Central Region of Ghana. The first phase determined the prevalence of *Plasmodium* infections in selected study sites. The second phase studied single nucleotide polymorphisms (SNPs) in the *P. falciparum Kelch 13*-domain (*PfKelch13*) in selected *Plasmodium*-positive samples, with the results reported by ecological zone and season.

### Selection of study areas and sampling period

An optimum allocation strategy was used to identify participating districts. Agona Swedru (AS), Assin Central (AC) and Gomoa East (GE) were from the forest ecological zone while Abura-Asebu-Kwamankese (AAK) and Cape Coast (CC) were from the coastal ecological zone. Participants were sampled systematically and concurrently in the respective district hospitals of the region.

Ecological features of the region: The study sites were classified into forest and coastal ecological zones based on the differences in environmental characteristics within the Central Region of Ghana. There are two rainy seasons each year in the area. The first is the major rainy season from April to July, with the heaviest rains in June. The second is the minor rainy season from September to November, with the heaviest rains in October. The vegetation cover comprises the coastal savanna, which extends about 15 km inland and parallels the Gulf of Guinea and the tropical rainforest, which is found in the inland areas toward the Ashanti and Eastern regional borders. Kakum National Park is a forest reserve in the district of Twifo Hemang Lower Denkyira, about 25 km from Cape Coast. Average temperatures in the area range from 21 to 31 °C, and annual rainfall ranges from 750 to 1,000 mm (Tormey and O'brien, 1993; Adams et al., 2017). Environmental characteristics of forest and coastal zones are displayed in **Table 1**. This ecological classification enabled us to assess possible differences in *PfKelch13* mutation profiles across different environmental settings. Participants were recruited in the rainy season (September and October 2020) and the dry season (November and December 2020).

**Table 1:** Environmental characteristics of forest and coastal zones.

Characteristic	Forest zone	Coastal zone
Vegetation	Predominantly moist deciduous forest	Coastal savanna vegetation
Rainfall	Generally higher and more reliable rainfall	Relatively lower and more seasonal rainfall
Ecosystem	Dense forest ecosystem with abundant vegetation	Coastal ecosystems with wetlands and mangroves
Landscape	More heavily vegetated and forested	More open landscapes with grassland and coastal vegetation
Key ecological features	Humid, Dense vegetation and forest cover	Wetlands, mangrove ecosystems, and coastal habitats
Location in the study area	Mainly the northern and central parts of the Central Region	Mainly the southern coastal part of the Central Region

### Criteria for study population selection

**Inclusion criteria:** Participant consent was critical to their inclusion. Evidence of slide-positive parasites with parasitaemia >500/ul and adequate haemoglobin for dried blood spot preparation were also considered.

**Exclusion criteria:** Participant enrolment was strictly contingent upon the provision of informed consent. Eligibility was further defined by microscopic evidence of *Plasmodium* infection with a parasite density exceeding 500 parasites/µL. Additionally, participants were required to have sufficient haemoglobin levels to facilitate the successful preparation of dried blood spots (DBS).

### Sample size determination and blood sample sampling procedure

Based on a previous malaria prevalence of 38.2% determined by microscopy (Dakorah et al., 2022), the required sample size was calculated using Cochran’s formula; n = z^2^p(1-p)/d^2^, where n = sample size, z = confidence level at 95% (standard value of 1.96), d = margin of error at 5% (standard value of 0.05); a sample size of 363 was arrived at; a minimum of 566 samples were collected.

### Participant recruitment and genomic sample selection

From each study site, hospital attendees were systematically recruited by selecting the first person at random, after which every 3rd patient who visited the hospital and consented to the study was enrolled. Initial malaria screening was conducted with RDT. Positive RDT cases were further subjected to microscopy to confirm the presence of *Plasmodium* parasites. Out of the microscopy-confirmed cases, samples were systematically selected for genomic study. The first sample was selected at random, after which every fifth malaria-positive slide (traced to sample) from each participating facility was selected for the preparation of dried blood spots.

### Clinical blood sample collection

Patients suspected to have malaria were enrolled after providing informed consent personally or through a legal guardian. An initial test for *P. falciparum* was carried out using the CareStart™ Malaria HRP2/pLDH COMBO Rapid Diagnosis Test (Access Bio, USA). On a positive RDT test, a 4 mL of venous blood sample was collected in EDTA tubes and homogenized. Parasitaemia was assessed via thick and thin blood film preparations according to WHO protocol. Thick films were prepared using 6 μL of blood, air-dried, stained with a 10% Giemsa solution for 10 minutes, and observed under a light microscope (Aninagyei, 2020).

### Dried blood spot preparation

Dried blood spots (DBS) were prepared in accordance with the Sanger Institute’s protocol by applying 50µL of whole blood to Whatman^®^ 3MM filter paper. The samples were air-dried at room temperature for 4 to 6 hours, ensuring protection from direct heat and sunlight. Following the drying process, individual cards were sealed in Ziplock bags containing a desiccant and maintained at ambient temperature until DNA extraction was initiated (Aninagyei et al., 2020).

### *Plasmodium* DNA Extraction

For DNA isolation, two to three 3-mm discs were excised from the dried blood spots using a sterile hole punch and transferred into sterile 1.5 mL microcentrifuge tubes. Genomic DNA was extracted with the QIAamp DNA Investigator Kit (Qiagen, CA, USA) following the manufacturer’s protocol. At least 5 ng of the resulting genomic DNA was utilized as the template for amplicon sequencing. To differentiate between *Plasmodium falciparum*, *P. vivax*, *P. knowlesi*, *P. malariae*, and *P. ovale*, species-specific primers were employed based on established protocols (Oyola et al., 2016; Aninagyei et al., 2020).

### Selective whole-genome amplifications (SWGA) of the *Plasmodium* genome

The selective whole-genome amplification (sWGA) methodology employed in this study followed a previously described stepwise protocol (Oyola et al., 2016). Custom-designed primers, typically 8–12 nucleotides in length, were utilised to preferentially target conserved, high-frequency motifs within the *P. falciparum* genome, specifically including the *PfKelch13* locus on chromosome 13. Isothermal amplification was performed using Phi29 DNA polymerase with a temperature step-down from 35 °C to 30 °C. The total reaction time was approximately 16 hours, with incubation intervals increasing from 5 minutes to the final 16-hour duration, in accordance with the original sWGA protocol and MalariaGEN standard operating procedures (Oyola et al., 2016; Aninagyei et al., 2020).

### Illumina amplicon sequencing using a HiSeq 2500 sequencer

Selective whole-genome amplification (sWGA) was performed using at least 5 ng of genomic DNA as the template. The reaction contained DNA template, 1× bovine serum albumin, 1 mM dNTPs, 2.5 μM of each primer, 1× Phi29 reaction buffer, and 30 units of Phi29 DNA polymerase (New England Biolabs). Amplification was performed under isothermal conditions with a stepwise decrease in temperature from 35 °C to 30 °C, followed by incubation at 30 °C for 16 h. The Phi29 polymerase was then inactivated at 65 °C. The amplified DNA was purified using Ampure XP beads, washed with 200 μL of 80% ethanol, and eluted in elution buffer. DNA libraries were subsequently prepared using the NEBNext^®^ Ultra™ DNA Library Preparation Kit (New England Biolabs) and sequenced on an Illumina HiSeq 2500 platform. Following sequencing, demultiplexing and generation of FASTQ files were performed automatically. Low-quality sequence reads were trimmed using BioEdit v7.2, and the resulting reads were analysed independently by mapping them to the *Plasmodium falciparum* 3D7 reference genome using the Burrows–Wheeler Aligner (BWA). Sequence reads corresponding to the *PfKelch13* locus were extracted for downstream analysis. Variants within the *PfKelch13* gene were identified by comparing mapped reads with the *P. falciparum* 3D7 reference sequence, with particular attention to nonsynonymous mutations previously associated with or implicated in artemisinin resistance. The identified *PfKelch13* variants were subsequently characterised according to their nucleotide and amino acid changes and their distribution among the study isolates (Oyola et al., 2016; Aninagyei et al., 2019; Aninagyei et al., 2020).

### Data processing and statistical analysis

Following data organisation in Microsoft Excel 2016, statistical analysis was carried out using SPSS Version 24 (Chicago, IL, USA). The prevalence of *PfKelch13* mutations across study sites was determined using frequency distributions and percentages. Pearson’s chi-squared tests were utilised to assess the relationship between these genetic markers and environmental factors, such as ecological classification (forest or coastal) and seasonality (dry or rainy). Multiple logistic regression analysis was conducted to investigate the effect of environmental factors influencing mutant haplotypes. P-values less than 5% were considered statistically significant.

### Ethical declaration

This study was conducted in strict adherence to the principles of the Declaration of Helsinki. Ethical approval for the research protocol was granted by the Ghana Health Service Ethics Committee (GHSERC017/03/20). All adult participants provided written informed consent prior to enrolment. For paediatric subjects, written informed consent was obtained from parents or legal guardians, supplemented by child assent where applicable for participants less than 19 years.

## Results

### Prevalence of malaria by study district

Of the 3,993 study participants suspected of malaria, 2,495 (62.5%) were screened using rapid diagnostic tests (RDTs). Among these participants, 1,525 (38.2%) were RDT-positive. The proportion of RDT-positive cases was highest in Agona Swedru Municipality (54.5%) and lowest in Abura-Asebu-Kwamankese District (20.0%). Sensitivity of clinical suspicion for malaria ranged from 58.2% in Abura-Asebu-Kwamankese District to 68.0% at Cape Coast Metropolis. In Abura-Asebu-Kwamankese District, Assin Central Municipality, Gomoa East District, Cape Coast Metropolis and Agona Swedru Municipality, the proportions of participants with suspected malaria who tested positive by RDT were 20.0%, 32.7%, 37.8%, 46.1% and 54.5% respectively (**Table 1**). Of 1,525 RDT-positive cases, microscopy confirmed Plasmodium parasites in 932 (61.1%). The numbers of samples analysed by study site were: Assin Central Municipality (190), Gomoa East District (143), Cape Coast Metropolis (95), Abura-Asebu-Kwamankese District (89), Agona Swedru Municipality (49) (**Table 2**).

**Table 2:** Distribution of Malaria by RDT and Microscopy.

Study facilities	Number of attendees	Number suspected of malaria n (%)	Number with malaria detected by RDT n(%)	Microscopy-confirmed malaria n (%)	Number selected for Genomic studies
AAK	529	182 (34.4)	106 (20.0)	62 (58.2)	89
ASM	660	613(92.8)	360 (54.5)	211 (58.7)	49
ACM	1203	676(56.2)	393 (32.7)	228 (58.1)	190
GED	872	530(60.8)	330 (37.8)	206 (62.3)	143
CCM	729	494(67.8)	336 (46.1)	228 (68.0)	95
Total	3993	2495(62.5)	1525 (38.2)	932 (61.1)	566

Data are presented as numbers and percentages (4). AAK, Abura, Asebu, Kwamankese District; ASM, Agona Swedru Municipality; CCM, Cape Coast Metropolis; GED, Gomoa East District; ACM, Assin Central Municipality.

The distribution of the 566 samples collected from the five study locations by season and ecological zone is shown in **Table 3**. Generally, sample collections were higher during the rainy season (399; 70.5%) than the dry season (167; 29.5%). The highest number of samples was recorded in the rainy season in Assin Central (136) and Gomoa East (140), while the lowest number of samples was recorded from Agona Swedru (15). The highest number of samples in the dry season was from Assin Central (54), followed by Abura-Asebu-Kwamankese (39) and Cape Coast (37). Three samples were collected at Gomoa East during the dry season, and a clear difference in sampling between the two seasons was observed at this site.

**Table 3:** Selected Genomic study samples distribution by Seasons and Ecological zones.

Variables	Abura-Asebu-Kwamankese	Agona Swedru	Assin Central	Cape Coast	Gomoa East	Total
Seasons						
Dry	39	34	54	37	3	167
Rainy	50	15	136	58	140	399
Total	89	49	190	95	143	566
Ecological Zones						
Coastal zone	69	20	62	74	26	251
Forest zone	20	29	128	21	117	315
Total	89	49	190	95	143	566

In relation to ecological zones, the forest zone comprised 315 samples (55.7%) and the coastal zone comprised 251 samples (44.3%). Thus, the forest zone contributed a somewhat higher proportion of the total samples. The forest zone was represented by the highest numbers from Assin Central (128) and Gomoa East (117). However, the coastal zone recorded the highest number of samples from Cape Coast (74) and Abura-Asebu-Kwamankese (69).

### Whole-genome amplification and sequencing of *PfKelch13* gene

Whole-genome amplification detected *Plasmodium* infections in 92.2% (522/566) of the five hundred and sixty-six (n = 566) samples selected for genomic studies, and amplification failed in 7.8% (44/566). These amplification failures could be caused by degradation of the parasite DNA or low numbers of parasites in the blood samples. A total of 358 *PfKelch13* genes (358/522, 68.6%) were successfully sequenced, and 164 (31.4%) were not successfully sequenced. Failures in sequencing may also be associated with insufficient quality or quantity of the amplification products. Such technical losses are known limitations of molecular surveillance studies from malaria samples collected from the field.

The wild-type (WT) *PfKelch13* allele remained the dominant allele across all sites of the study (42% to 100%), although the burden and diversity of mutant alleles varied widely by site. Of the identified mutations, single mutations were found in 127 patients. Double mutations (n = 42) were less common than single mutations but displayed site-specific enrichment where present. Triple mutations were the least frequent (n = 2), and a quadruple mutation was found in one subject (n = 1). The analysis was restricted to single-nucleotide polymorphisms (SNPs) that lead to amino acid substitutions in the *PfKelch13* propeller domain (codons 436–706). Synonymous variants were not identified in the sequence dataset and therefore were not included in the mutation profiling analysis. **Figure 1** shows the distribution of SNPs by study site.

**Figure 1:**
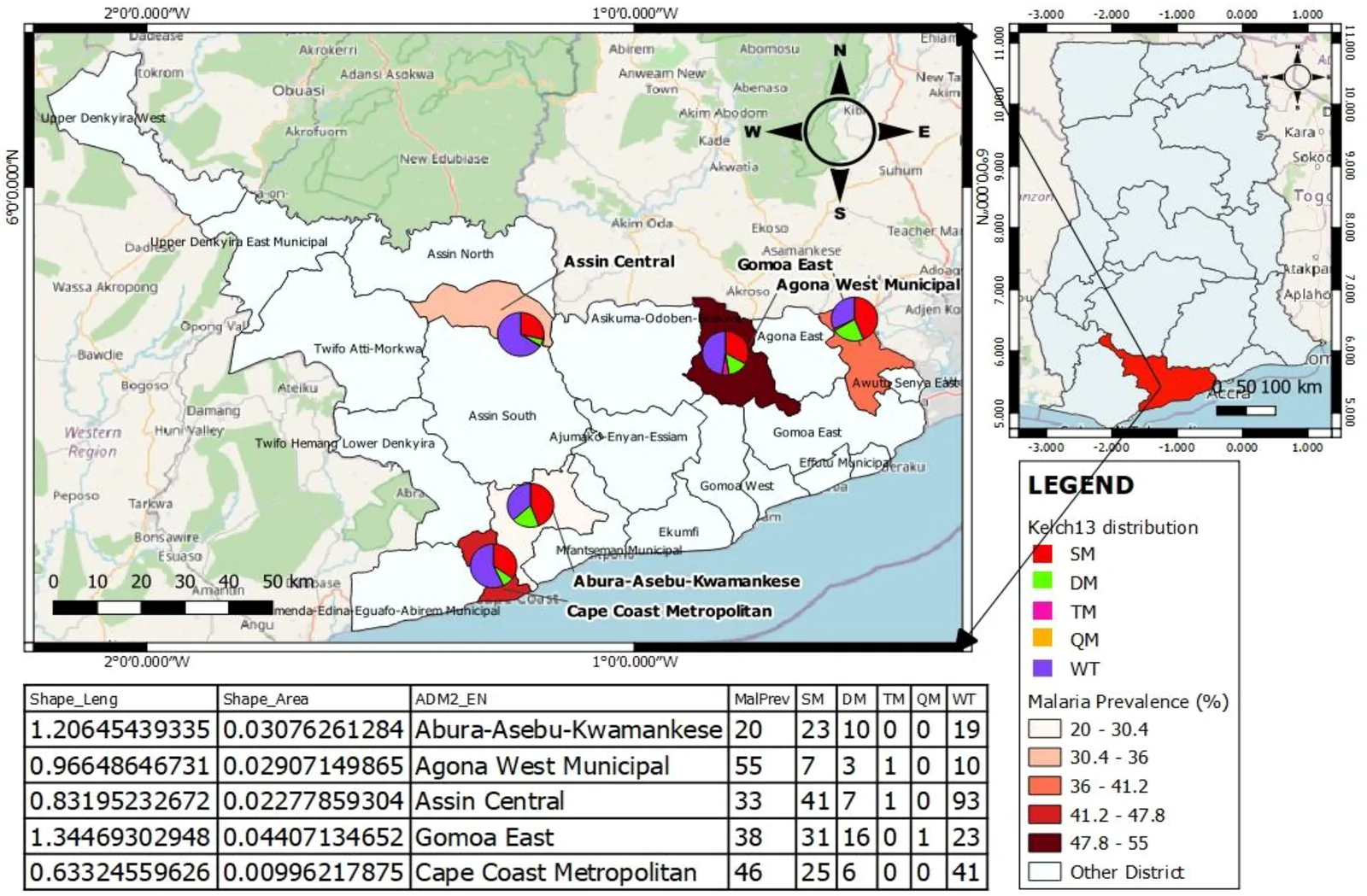
Spatial mapping of prevalence of malaria and *PfKelch13* distribution in the selected districts in the Central Region of Ghana. Key: SM, Single Mutation; DM, Double Mutation; TM, Triple Mutation; QM, Quadruple Mutation; WT, Wild Type.

### Distribution of mutations observed in the *PfKelch13* gene in *P. falciparum* mutations per District

**Figure 2** shows the distribution of mutations observed in the *P. falciparum PfKelch13* gene in districts. The majority of the mutations were concentrated at A578V (31, 8.66%), P413L (27, 7.54%), A676D (18, 5.03%), A578S (12, 3.35%) and S485G (6, 1.68%). The double mutation A578V–D516G (4.75%) was the most common multiple mutation; it was present in 17 samples and was present in all districts except the Assin Central Metropolis. The validated *PfKelch13* artemisinin resistance marker M476I and the candidate marker N537D were found in the double mutations A621D–M476I and A578V–N537D, respectively. The validated marker was found in the Assin Central Metropolis, while the candidate marker was identified in the Cape Coast Metropolis sample. Variants of both the validated and candidate markers were also found at very low frequencies (<1%). Triple mutations were found in isolates from Assin Central and Agona West, and the only isolate with a quadruple mutation was from a sample in Gomoa East.

**Figure 2:**
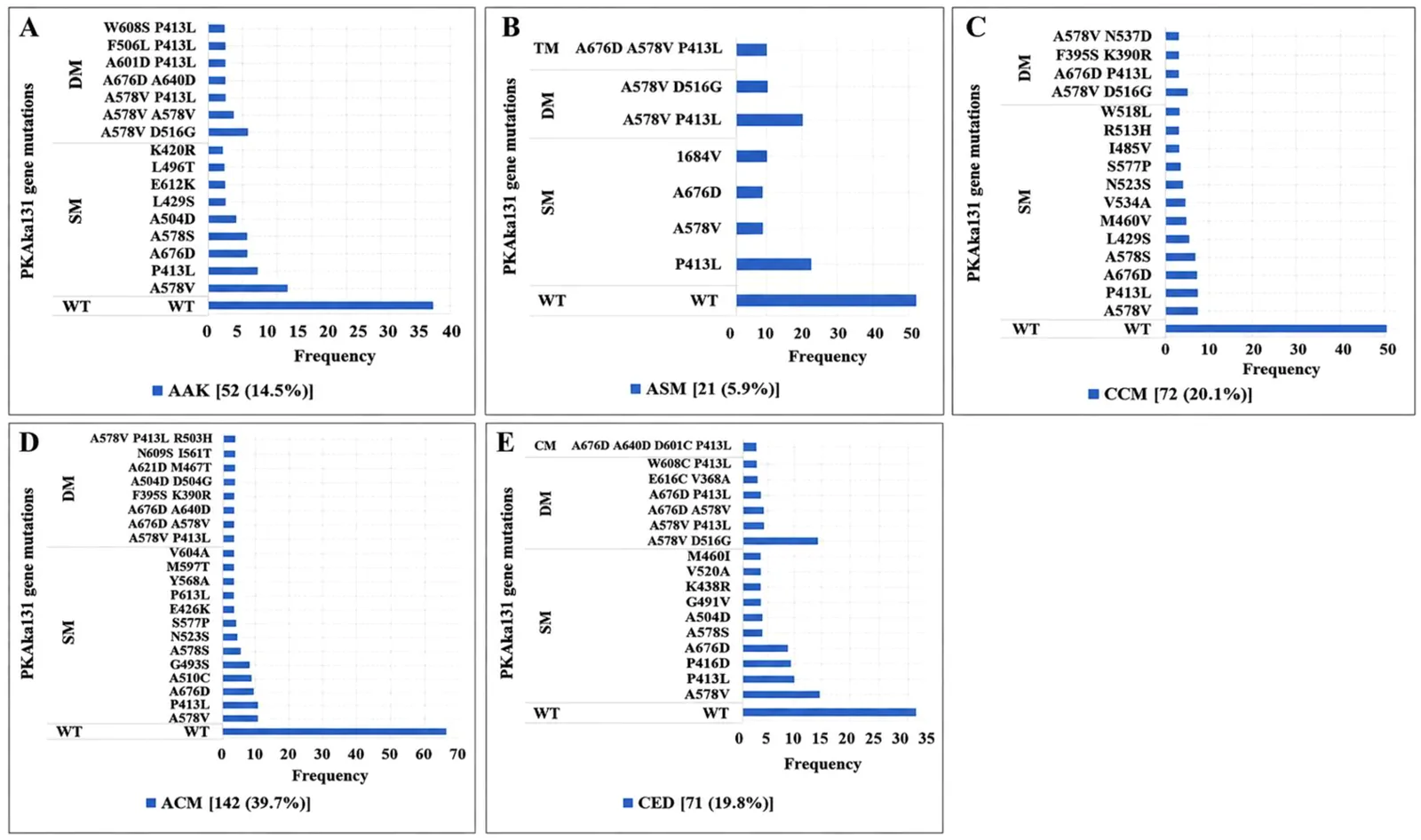
Distribution of mutations observed in the *PfKelch13* gene in *P. falciparum* per District. Key: **(A)** Abura Asebu Kwamankese, **(B)** Agona Swedru Metropolis, **(C)** Cape Coast Metropolis, **(D)** Assin Central Metropolis, **(E)** Gomoa East District.

AAK had the lowest rate of WT isolates (42%) among the sites, indicating a relatively high mutation burden. The most frequent mutation was A676D (11%), followed by A578V-D516G (8%), A578S (8%) and the double mutation A676D-A578V (8%). Other mutations detected were A578V (7%), P413L (4%), A578V–P413L (4%), A504D (4%) and K420R (4%). ASM was characterised by a higher percentage of WT isolates (53%) and a narrower mutation spectrum. Mutations were fairly evenly distributed at low frequencies, with A676D-A578V-P413L (7%), I684V (7%), P413L (7%), A676D (7%) and A578V (6%) as the most frequently observed variants.

WT isolates accounted for 44% of the samples in CCM. The most common mutation was P413L (13%), followed by A578V (9%) and the double mutation A578V–D516G (9%). Other mutations were found at lower frequencies, such as A676D (5%), N523S (4%), R513H (4%), V534A (4%), M460V (4%) and L429S (4%). ACM was associated with the highest proportion of WT isolates (66%) among the mutation-carrying sites, but also with the broadest spectrum of low-frequency variants. The most common mutation was A578V (9%), followed by S485G (7%), A676D (4%) and P413L (4%). Several rare mutations were observed at 2% frequency, including A621D, M579T, S577P, A504D–D501G and A578V–P413L–R393I. However, GED recorded wild type (100%) and no *PfKelch13* mutations were identified.

### Ecological distribution of single mutation in *PfKelch13* by study district and ecological zones

Ecological stratification revealed clear differences in the distribution of single *PfKelch13* mutations between the Coastal and Forest zones. Of the 358 isolates analysed, 172 (48.04%) had single mutations, with a greater absolute number in the Forest ecological zone (n=108/234; 46.15%) than in the Coastal zone (n=64/124; 51.61%). However, the rate of single-mutation isolates was higher in the Coastal zone (51.61%) than in the Forest zone (46.15%). The wild-type was slightly more prevalent in the Forest zone (53.85%) than in the Coastal zone (48.39%). The Forest zone had higher mutation diversity and higher frequencies of some variants, particularly A578V, P413L, and A676D, as well as a broader diversity of low-frequency mutations. However, there was a statistically significant association between ecological zone and the distribution of observed mutations (χ2(186) = 250.04; *p* = 0.001) (**Table 4**).

**Table 4:** Ecological distribution of single mutation in *PfKelch13* by study district and ecological zones.

Amino acid locus	Overall Total N = 358		Coastal sone N = 124			Forest zone N = 234			
	Freq.	Percent	Abura-Asebu-Kwaman- kese n=52	Cape Coast n=72	Total	Agona Swedru n=21	Assin Central n=142	Gomoa East n=71	Total
Wild type									
WT	186	51.96	19	41	60	10	93	23	126
Single									
A578V	31	8.66	7	4	11	1	9	10	20
P413L	27	7.54	4	4	8	4	9	6	19
A676D	18	5.03	3	3	6	1	6	5	12
A578S	12	3.35	3	3	6	–	1	5	6
S485G	6	1.68	–	–	–	-	6	–	6
A504D	3	0.84	2	-	2	-	-	1	1
A621D	3	0.84	-	–	–	–	3	-	3
L429S	3	0.84	1	2	3	–	–	–	–
S577P	2	0.56	–	1	1	–	1	–	1
M460V	2	0.56	–	2	2	–	–	–	–
N523S	2	0.56	-	1	1	-	1	-	1
V534A	2	0.56	–	2	2	–	–	–	-
E426K	1	0.28	–	–	–	–	1	–	1
E612K	1	0.28	1	–	1	–	–	–	–
F614S	1	0.28	–	–	–	–	1		1
G591V	1	0.28	–	–	–	–	–	1	1
I405V	1	0.28	-	1	1	–	–	–	–
I406T	1	0.28	1	–	1	–	–	–	–
I684V	1	0.28	–	–	–	1	–	–	1
K420R	1	0.28	1	–	1	–	–	–	–
K438R	1	0.28	–	–	–	–	–	1	1
M460I	1	0.28	–	–	–	–	–	1	1
M579T	1	0.28	–	–	–	–	1	–	1
R513H	1	0.28	-	1	1	–	–	–	–
V520A	1	0.28	–	–	–	–	–	1	1
V568A	1	0.28	–	–	–	–	1	–	1
V603A	1	0.28	–	–	–	–	1	–	1
W518L	1	0.28	–	1	1	–	-	–	-

### Distribution of multiple mutations in *PfKelch13* by study district and ecological zones

Multiple mutations were observed in 34 isolates (9.50%), with a slightly higher proportion in the Forest ecological division (n = 23/234; 9.83%) than in the Coastal division (n = 11/124; 8.87%). Overall, double mutations were the most common (91.2% of isolates with multiple mutations), with A578V_D516G being the most common combination (4.75% overall). The Forest zone encompassed all triple- and quadruple-mutation profiles and the majority of double-mutation combinations. By contrast, the Coastal zone showed sporadic combinations of double mutations and no higher-order mutation patterns. Some mutations were common to both the Coastal and Forest zones, while some were found mainly in one zone. Statistically significant association was found between the ecological zone and the observed mutations (χ2(186) = 250.04; *p* = 0.001). **Tables 4** and **5** present the mutations.

**Table 5:** Distribution of multiple mutations in *PfKelch13* by study district and ecological zones.

Amino acid locus	Overall Total N = 358		Coastal zone N = 124			Forest zone N = 234			
	Freq.	Percent	Abura-Asebu-Kwamankese n=52	Cape Coast n=72	Total	Agona Swedru n=21	Assin Central n=142	Gomoa East n=71	Total
Double mutation									
A578V D516G	17	4.75	3	3	6	1	-	10	11
A578V P413L	5	1.4	1	–	1	2	1	1	4
A676D A578V	4	1.12	2	–	2	–	1	1	2
A676D P413L	3	0.84	–	1	1	–	–	2	2
A676D A504D	2	0.56	1	–	1	–	1	–	1
F395S K390R	2	0.56	–	1	1	–	1	–	1
A504D P413L	1	0.28	1	-	1	–	-	–	-
A578V N537D	1	0.28	-	1	1	–	–	–	–
A621D M476I	1	0.28	–	–	–	–	1	–	1
E651G V356A	1	0.28	–	–	–	–	–	1	1
F506L P413L	1	0.28	1	–	1	–	–	–	–
N609S I551T	1	0.28	–	–	–	–	1	–	1
W660C P413L	1	0.28	–	–	–	–	–	1	1
W660S P413L	1	0.28	1	–	1	–	–	–	–
Triple mutation									
A578V P413L R393I	1	0.28	–	–	–	-	1	–	1
A676D A578V P413L	1	0.28	–	–	–	1	-	–	1
Quadruple Mutation									
A676D A504D D501G P413L	1	0.28	–	–	–	–	–	1	1

### Seasonal distribution of the top ten (10) *PfKelch13* gene mutations observed in both dry and rainy seasons per district

**Figures 3** and **4** show the seasonal distribution of the top ten (10) *PfKelch13* gene mutations detected in the dry and rainy seasons in the different districts. The WT frequencies were generally lower during the rainy season at all sites, and the reductions were especially significant at AAK, ASM and GED. In AAK, WT decreased from 42% in the dry season to 35% in the rainy season, with large increases in A578V (7%→22%) and P413L (4%→13%) and the double mutation A578V–D516G was detected. During the wet season, the ASM displayed a significant seasonal shift in the mutation profile relative to the dry season, with WT dropping from 53% to 33%, P413L rising dramatically from 7 to 50%, and A578V–D516G emerging prominently (17%). The most prominent difference was at GED, where isolates were all WT in the dry season (100%), but large diversification occurred in the rainy season, when WT decreased to 35%, and A578V, A578V-D516G, P413L, and A676D increased to high frequencies of 16%, 16%, 9% and 8%, respectively. Conversely, CCM and ACM showed relatively higher seasonal stability. In both seasons, the proportion of WT was relatively high in CCM (44% in the dry season vs. 71% in the rainy season). WT isolates were present at high frequency in ACM, but with a broad spectrum of mutations, at low frequency and across seasons (66% in the dry season versus 71% in the rainy season), indicating little or no seasonal amplification of particular variants.

**Figure 3:**
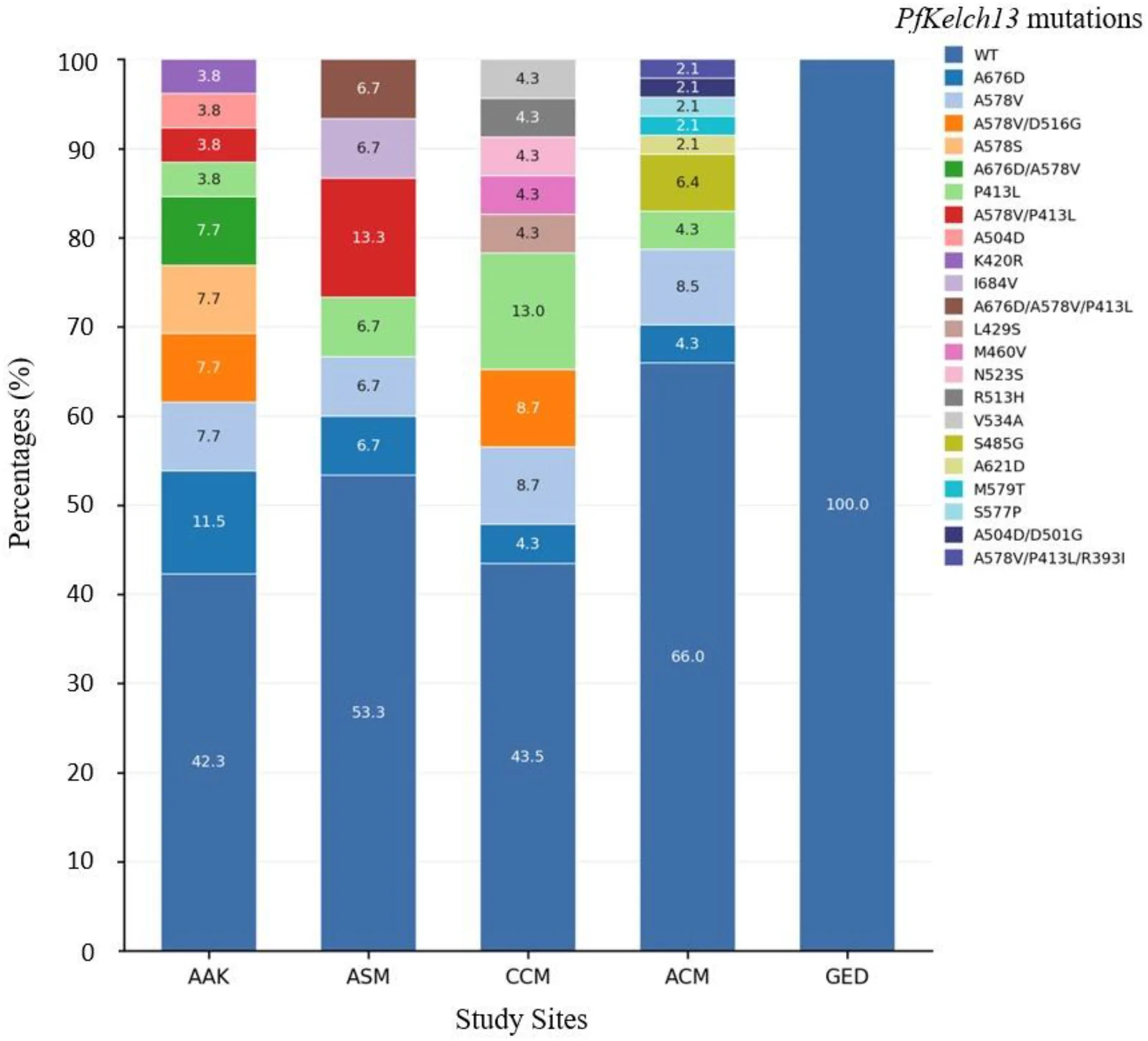
Distribution of the ten most frequently observed *PfKelch13* mutation profiles observed in the Dry Season by district. Key: **(A)** Abura Asebu Kwamankese, **(B)** Agona Swedru Metropolis, **(C)** Cape Coast Metropolis, **(D)** Assin Central Metropolis, **(E)** Gomoa East District. The selection of top ten mutations were for data presentation and visual clarity and not for the statistical exclusion of other mutations.

**Figure 4:**
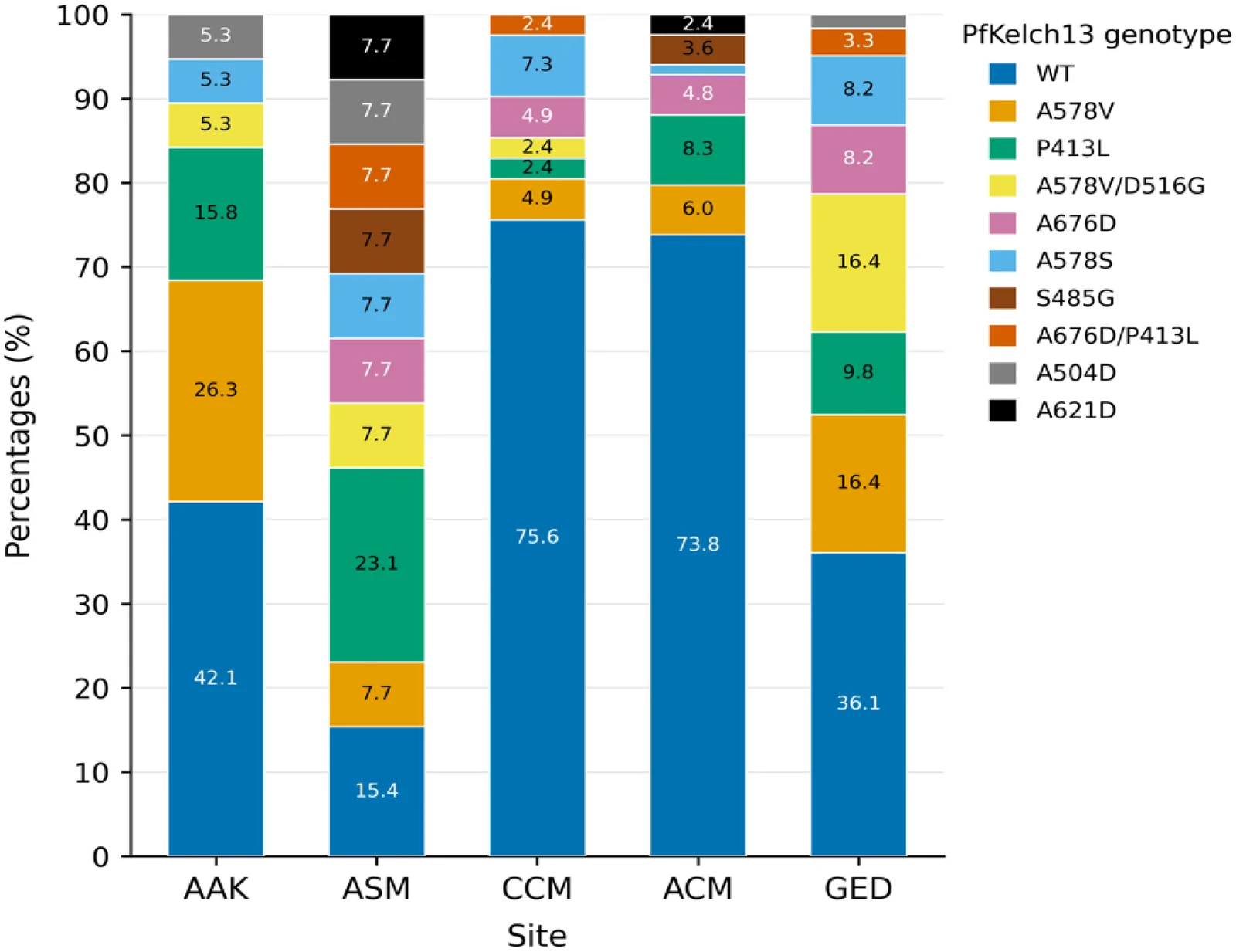
Distribution of the ten most frequently observed *PfKelch13* mutation profiles observed in the Rainy Season per district. Key: **(A)** Abura Asebu Kwamankese, **(B)** Agona Swedru Metropolis, **(C)** Cape Coast Metropolis, **(D)** Assin Central Metropolis, **(E)** Gomoa East District. The selection of top ten mutations were for data presentation and visual clarity and not for the statistical exclusion of other mutations.

### Environmental factors associated with *Plasmodium falciparum* mutant haplotypes

**Table 6** presents multivariable logistic regression analysis results of associations between selected environmental and spatial factors and presence of mutant *PfKelch13* haplotypes. The model had independent variables of ecological zone, season and district, with reference categories being coastal ecological zone, dry season and Abura Asebu Kwamankese District, respectively. Odds ratios (ORs) and 95% confidence intervals (CIs) are given. When controlling for season and ecological zone, district was the only environmental factor that was consistently associated with selected mutant haplotypes. Compared with the reference district (Gomoa East), participants from Assin Central had significantly lower odds of A578V (OR = 0.22, 95% CI: 0.08–0.62, *P* = 0.004), A676D (OR = 0.25, 95% CI: 0.06–0.99, *P* = 0.048), and A578S (OR = 0.06, 95% CI: 0.01–0.56, *P* = 0.014). Only Model I (A578V) (*P* = 0.019) and Model IV (A578S) (*P* = 0.020) were statistically significant at the overall model level. Models II, III, and V were not statistically significant. Model IV had the highest pseudo-R^2^ (0.147) and the best discrimination performance with an AUC of 0.784, indicating acceptable discrimination. The AUCs of Model I, Model II, Model III and Model V were 0.687, 0.633, 0.631 and 0.707, respectively.

**Table 6:** Multiple logistic regression analysis of environmental factors associated with *PfKelch13* mutant haplotypes.

Environmental factor	Model I: A578V		Model II: P413L		Model III: A676D		Model IV: A578S		Model V: A578V D516G	
	OR (95% CI)	*P*	OR (95% CI)	*P*	OR (95% CI)	*P*	OR (95% CI)	*P*	OR (95% CI)	*P*
Ecological zone										
Coastal zone	Reference	—	Reference	—	Reference	—	Reference	—	Reference	—
Forest zone	1.21 (0.34–4.36)	0.771	0.99 (0.22–4.50)	0.994	1.88 (0.40–8.82)	0.424	0.96 (0.21–4.46)	0.962	3.16 (0.54–18.55)	0.202
Season										
Dry season	Reference	—	Reference	—	Reference	—	Reference	—	Reference	—
Rainy season	0.95 (0.35–2.56)	0.916	1.63 (0.52–5.09)	0.401	0.61 (0.21–1.75)	0.361	2.04 (0.47–8.91)	0.341	0.79 (0.13–4.82)	0.795
District										
Gomoa East	Reference	—	Reference	—	Reference	—	Reference	—	Reference	—
Agona West Municipal	0.22 (0.02–2.14)	0.192	2.04 (0.40–10.41)	0.389	0.31 (0.03–3.29)	0.331	1	—	0.19 (0.01–4.02)	0.289
Cape Coast Metropolitan	0.27 (0.06–1.11)	0.069	0.41 (0.08–2.10)	0.286	0.56 (0.11–2.92)	0.491	0.36 (0.07–1.76)	0.208	0.50 (0.07–3.38)	0.478
Assin Central	**0.22 (0.08–0.62)**	**0.004**	0.42 (0.13–1.33)	0.141	**0.25 (0.06–0.99)**	**0.048**	**0.06 (0.01–0.56)**	**0.014**	1	—
Model statistics										
Observations	217		213		204		188		110	
Wald χ²	13.46		6.79		4.55		11.71		8.02	
Model *P*-value	**0.019**		0.237		0.473		**0.020**		0.091	
Pseudo R²	0.071		0.046		0.039		**0.147**		0.086	
AUC	0.687		0.633		0.631		**0.784**		0.707	

Statistically significant at *P*-value < 0.05*.

OR, odds ratio; CI, confidence interval; AUC, area under the receiver operating characteristic curve.

## Discussion

The emergence of drug-resistant Plasmodium falciparum has been one of the greatest challenges to malaria control in the past 50 years, defining the global health agenda and requiring periodic revision of treatment guidelines (Nzoumbou-Boko et al., 2020). Several validated and candidate markers in the *PfKelch13* gene so far have been reported from different geographic regions (Matrevi et al., 2019; Ikegbunam et al., 2022). In this study, we explored molecular markers in *P. falciparum* populations from clinical samples collected in selected districts of the Central Region of Ghana. This cross-sectional survey focused on identifying mutations and previously validated drug-resistant markers in the *PfKelch13* gene in *P. falciparum*.

One of the most striking findings of our genome sequencing was the observation of mutations in *PfKelch13* at 48% of the sites that we studied. This is a substantial increase as compared to other regional estimates in Ghana, where mutation rates of 16% and 16.6% have been previously reported (Matrevi et al., 2019; Aninagyei et al., 2020). These findings were also higher than the prevalence of 36% (25/72) reported by Dieng et al. in their study in the northern region of Ghana (Dieng et al., 2023). This observed genetic diversity is also consistent with the previously described diversity of *PfKelch13* polymorphisms in sub-Saharan Africa (Kamau et al., 2015). This pattern has been further supported by molecular epidemiological studies across the continent, which have identified novel mutations not reported in other studies or in Southeast Asia (de Laurent et al., 2018; Ajogbasile et al., 2022; Rodrigues et al., 2022). The differences between studies may be due to geographical variation, sampling times, parasites studied and methodological differences (Cheeseman et al., 2012).

Observed mutations exhibited variable codon-level polymorphism, with affected codons containing 1–4 single nucleotide polymorphisms (SNPs). Double mutations accounted for 24.4% of all detected mutations. Especially, these were the validated marker combination (M476I-A621D) and the candidate combination (N537D-A578V), with a low prevalence of 0.28%. Interestingly, this is not the first time that a validated marker has been found in isolates from Ghana. The M476I mutation was first identified in a sample collected from Wa in the Upper West Region of Ghana (Matrevi et al., 2019). The Y493H marker was previously reported by the MalariaGEN surveillance program (MalariaGen and Plasmodium falciparum Community Project, 2016). A validated marker, C580Y, was subsequently identified in *P. falciparum* isolates from neighbouring Greater Accra and Ashanti regions of Ghana (Aninagyei et al., 2020; Dieng et al., 2023). F446 was detected in two samples in a study of molecular markers of drug resistance in imported cases of malaria (Zhao et al., 2021). On the contrary, the Y493H, R561H, P553L, C580Y, and F446I were not detected in our study area but were confirmed in previous studies from Ghana and other parts of the continent (Owoloye et al., 2021; Chaves et al., 2024). Among the observed variants, A578V, P413L, A676D and A578S are mostly polymorphisms of *PfKelch13*, and their association with artemisinin resistance is unknown.

In addition to the double mutants, we also found codons that were mutated three and four times in our analysis. Several *PfKelch13* substitutions indicate the genetic diversity of the parasite populations, but the functional consequence of such combinations remains unknown. These multiple mutations may have epistatic effects, being more resistant or less fit. This type of interplay has been suggested in previous studies of complex mutant strains where multiple mutations may interact to affect parasite fitness, survival and transmission (Mbengue et al., 2015; Stokes et al., 2021; Behrens et al., 2022). Considering the high infection rate in our previous studies (Dakorah et al., 2022), the relatively high mutation rate in the present study, the low prevalence of validated markers and the rapid emergence of artemisinin resistance in Africa (Ndwiga et al., 2021; Gashema et al., 2025), it would be worth exploring this further by means of therapeutic efficacy studies (TES). Such studies may help to elucidate the functional roles of the observed SNPs and whether particular mutations or combinations of mutations have a synergistic contribution to resistance. This evidence will be essential in guiding timely case management and overcoming resistance strategies.

Differences in genetic diversity, distribution of variants and prevalence of wild-type (WT) parasites were observed between the Forest and Coastal ecological zones for genetic variation across the different ecological divisions. The Forest zone had higher total variant load, higher frequency of rare variants and more pronounced WT reservoir features compared to the Coastal zone. The Coastal zone had lower genetic diversity but retained several important mutations at low to moderate frequencies.

Mutational profiles were different among ecological zones, but after adjustment, ecological classification was not significantly associated with mutant haplotypes. The relatively low number of WT parasites relative to the number of variant observations in the Coastal zone may indicate more dynamic transmission pressures, including environmental variability and increased human movement, which may enable periodic introductions and redistribution of parasite variants. This pattern is consistent with ecological contexts that would permit sustained cycles of transmission, possibly resulting in gradual accumulation and maintenance of genetic diversity over time (Mosseler et al., 2003; Stoddard et al., 2009; Salmona et al., 2023).

Genomic analysis revealed an excess of rare variants in the overall parasite population, with a high number of singleton variants and a low number of alleles at high frequency. This pattern is consistent with mutation-selection dynamics and limited fixation of dominant variants under rather moderate selection pressures across the continent (MalariaGen and Plasmodium falciparum Community Project, 2016). Sites such as AAK and CCM showed evidence of mutation accumulation, including double and triple substitutions. The AAK pattern is attributable to a genetically heterogeneous population of parasites in which both single and double mutations contribute significantly to the overall profile. CCM exhibited moderate genetic diversity with enrichment of P413L and considerable contribution of a resistance-associated double mutation. The ASM was not very diverse and balanced, but the relatively narrow distribution of mutations suggested low selective pressure and no expansion of one dominant variant. This profile of ACM indicated a mutational reservoir of rare variants, rather than the expansion of specific high-frequency mutations. GED was totally wild type, indicating marked differences in evolutionary or drug selection pressure between study sites. This result is in agreement with the variation in the degree of selection pressure and environmental niches (Matrevi et al., 2022; Dieng et al., 2023).

We observed a slightly higher distribution of *PfKelch13* haplotypes in the rainy season, but overall frequencies were similar in both seasons (53.2% dry season vs 51.3% rainy season). This shows that the WT haplotype was relatively stable over the year and that a baseline level was maintained throughout both transmission periods. WT proportion varied between 42% and 100% during the dry season, and between 33% and 71% during the rainy season, indicating higher mutational burden in the rainy season. Data from the rainy season also showed greater mutational complexity and less WT dominance at some sites, in particular AAK, ASM and GED, than in the dry season. The emergence and spread of A578V, P413L and A578V-D516G during this period indicate that transmission dynamics are a factor in the selection and maintenance of *PfKelch13* mutations through the seasons. Conversely, CCM and ACM had similar genetic profiles, which would be consistent with site-specific stability, even with the different selective pressures between dry and rainy seasons. Similar variations have been reported by Adjah et al. (2018).

Here, we provide evidence of seasonal variation in the mutation profiles of *PfKelch13* with a descriptive increase in mutational burden and diversification at several sites during the rainy season. However, season was not independently associated with mutant haplotypes in the regression analysis. Determining whether seasonal transmission dynamics drive variation in *PfKelch13* would require longitudinal studies. Sites such as ASM and GED, which showed marked seasonal shifts, may represent environments in which selective forces are not constant but episodic, and may allow the transient expansion of advantageous variants during periods of high transmission, as described elsewhere (Reiner et al., 2015; Guery et al., 2025; Tantuoyir, 2025). In contrast, the relative stability at CCM and ACM may reflect ongoing transmission with little directional selection or effective buffering of parasite population dynamics (Mwesigwa et al., 2025). The observed spatial differences suggest heterogeneity of *PfKelch13* profiles across districts and warrant further investigation.

Of particular interest is the emergence of multiple mutations during the rainy season, especially A578V-D516G, as it represents an example of patterns of genetic variation that may occur under seasonal drug-selection pressures, even where resistance-associated mutations have not been fixed. These mutations do not necessarily confer clinical resistance, but some *PfKelch13* variants may have functional consequences on artemisinin susceptibility and need further investigation (Stokes et al., 2021). The lack of mutations at GED in the dry season and high diversification in the rainy season point to the importance of seasonality in molecular surveillance, since sampling in a single season can underestimate the underlying genetic diversity and potential resistance landscape. These results suggest that seasonality could be a key factor in the evolution and distribution of markers associated with antimalarial resistance.

In the multivariable analysis (**Table 6**), an apparently counterintuitive pattern emerged, with district of residence rather than ecological zone or season being the environmental variable most consistently associated with carriage of *PfKelch13* mutant haplotypes. In Ghana, molecular surveillance has demonstrated that ecological zone and season could be proxies for transmission intensity, entomological inoculation rate, and level of naturally acquired antimalarial immunity, all of which have traditionally been thought to be relevant to the selection and spread of drug resistance alleles under a given level of drug pressure (Tandoh et al., 2026). This framework was explicitly applied in a recent national amplicon deep-sequencing study reporting significantly different entomological inoculation rates across ecological zones (approximately ten-fold higher in the Forest and Savanna zones compared to approximately 20 in the Coastal zone). Validated *PfKelch13* mutations were more common in the Forest zone, not in the lower-transmission Coastal zone predicted by the classical model. Temporal and zonal trends across resistance genes were not statistically significant for most of the genes (Tandoh et al., 2026).

The association of selected environmental and spatial factors with the presence of mutant *PfKelch13* haplotypes was evaluated through multivariable logistic regression analysis. Descriptive analyses supported seasonality, but the effect was attenuated in adjusted models, and several districts had significantly different odds of carrying the mutation. The strong signal for A578V, A676D and A578S being district-level and not ecological zone or season is broadly in agreement with Tandoh’s study. General ecological types and descriptive seasonal differences may therefore not be able to explain the distribution of mutant haplotypes at finer spatial and temporal scales. Similarly, a study from the Bongo District in the Upper East Region of Ghana found that the asymptomatic parasite reservoir did not exhibit a clear geospatial population structure, but there was significant linkage disequilibrium between resistance loci. Thus, the drug-resistance haplotypes in Ghana may be acting in a locally specific way that does not map well onto general ecological or seasonal categories (Narh et al., 2020).

This finding, however, is consistent with the absence of a significant geographical association reported by Dieng and colleagues, suggesting a more complex interpretation is needed than simple ecological zones, although they did not use logistic regression (Dieng et al., 2023). Similarly, another study reported high parasite diversity in all ecological zones with heterogeneous connectivity between zones, suggesting that simple ecological classification may not fully capture the spatial structure of parasite genotypes (Amoah et al., 2021). Taken together, these findings suggest that local factors at the district level, such as district-specific treatment practices, access to antimalarial drugs, transmission intensity, movement of populations, and health-system performance, may have stronger or more immediate selective effects than macro-ecological classifications in determining the distribution of mutant haplotypes. The large inter-district differences demonstrate the utility of geographically stratified molecular surveillance.

Importantly, the mutations included in the regression analysis were A578V, A676D and A578S. They were selected because of their frequency in the present study. Specifically, A578S has been well characterised in Ghanaian *P. falciparum* populations: archived isolates collected from 2007 to 2016 showed a low national prevalence of this mutation (Matrevi et al., 2019) and it is still detected at low frequencies in subsequent site-level and nationwide surveys, with higher local prevalence reported in some ecological zones and years (Matrevi et al., 2022; Matrevi et al., 2024; Kegya et al., 2026; Tandoh et al., 2026).

The presence of A578S, A578V and other similar propeller domain substitutions is more consistent with ongoing background diversification of the *PfKelch13* gene under continued drug selection rather than confirmation of clinical resistance, since they are not part of the validated list of markers of artemisinin partial resistance by the World Health Organisation. In Ghana, local studies have identified several *PfKelch13* polymorphisms, including novel and site-restricted variants, which appear to be associated with low nucleotide diversity and purifying selection, contrasted with the clonal expansion of validated resistance alleles observed in some other parts of Africa (Owoloye et al., 2021; Matrevi et al., 2022). Thus, the district-specific reduction in odds of carrying these haplotypes in Assin Central relative to Gomoa East may be more sparingly explained by differences in localised parasite population structure and drug exposure history rather than direct evidence of differential resistance risk.

Several aspects of the regression results suggest caution in interpreting the district-level associations. Only two of the five haplotype-specific models (models I and V) were significant overall. The best model for A578S explained only a small proportion of the variance (pseudo-R^2^ = 0.147), but had acceptable discrimination (AUC = 0.784). The wide confidence intervals for some odds ratios, in particular for A578S (95% CI 0.01-0.56), are likely due to the low absolute prevalence of these haplotypes and small counts in some strata. Similar limitations have been reported for rare *PfKelch13* variants in other molecular surveillance datasets from Ghana (Matrevi et al., 2019; Matrevi et al., 2022; Kegya et al., 2026; Tandoh et al., 2026). The insignificance of Models II-IV also indicates that the estimated relationships are especially uncertain. Although districts do seem to capture real local variation, the underlying drivers were not directly measured in this analysis, and so cannot be disentangled from district identity per se. Thus, associations at district level should be taken as indicative of spatial heterogeneity and not as conclusive evidence for the independent contribution of district-specific characteristics to the distribution of *PfKelch13* mutations.

## Study limitations

A limitation of this study was the inability to resolve the complex polyclonal haplotypes into individual parasite clones. This was because the amino acid calls detected at each gene locus of the target genes were provided by the Malaria Genome Laboratory at the Wellcome Sanger Institute rather than the underlying sequence reads. Second, the putative drug-resistant haplotypes reported here cannot be directly linked to chloroquine or sulfadoxine/pyrimethamine resistance, because their therapeutic significance was not determined. Finally, 158 (30.3%) of the 522 parasites were not sequenced, and the reasons for failure in sequencing were not studied.

## Conclusion

Artemisinin-based combination therapy (ACT) is a highly effective treatment for malaria. The emergence and spread of *P. falciparum* parasites with reduced susceptibility to ACTs threatens malaria control efforts. While mutations in *PfKelch13* are prevalent in Southeast Asia, they are also present at low frequencies in African countries, such as Ghana. The most common single nucleotide polymorphisms (SNPs) identified in our study were the *PfKelch13* mutations A578V, P413L, A676D, A578S, and S485G. These mutations, with high mutational burden, may play a role in the genetic background that favours the emergence or persistence of *PfKelch13*-associated resistance mutations. We detected the validated artemisinin resistance marker M476I and the candidate marker N537D, but at very low frequencies, suggesting limited current selection for established artemisinin resistance. Interestingly, only a handful of isolates harboured double, triple or quadruple *PfKelch13* mutations, perhaps a reflection of the destabilising effects that multiple mutations may have on parasite fitness and viability.

Functional characterisation of newly reported SNPs, including those identified in this study, is important because artemisinin resistance may arise independently in different ecological zones. The clear spatial heterogeneity in mutation patterns and distribution of wild-type parasites highlights localised evolutionary dynamics and reinforces the need for continuous, site-specific molecular surveillance. Thus, there is an urgent need to monitor artemisinin resistance and ACT treatment failure through integrated clinical, epidemiological and genomic surveillance. These efforts may also improve early detection, surveillance, and containment of artemisinin resistance and ACT treatment failure and allow for the integration of local genomic data into global databases to track the emergence and spread of antimalarial drug resistance.

The spatial and temporal heterogeneity of forest, coastal and savanna transmission settings suggests that factors operating at finer geographical and temporal scales may influence genetic variation. The routine molecular surveillance with district-level stratification could thus improve the evidence-based refinements of malaria control strategies and reinforce the importance of geographically granular surveillance as the foundation of effective artemisinin resistance monitoring and containment. Future studies with larger sample sizes at the district-level, historical data on drug pressure, and population-genetic metrics would help to determine whether the observed district-level effect in this study reflects real differences in local selective pressure or stochastic sampling variation associated with a low-prevalence outcome.

## Data Availability

The datasets presented in this study can be found in online repositories. The names of the repository/repositories and accession number(s) can be found in the article/supplementary material.
